# Future roots for future soils

**DOI:** 10.1111/pce.14213

**Published:** 2021-11-29

**Authors:** Jonathan P. Lynch, Sacha J. Mooney, Christopher F. Strock, Hannah M. Schneider

**Affiliations:** ^1^ Department of Plant Science The Pennsylvania State University University Park Pennsylvania USA; ^2^ School of Biosciences University of Nottingham Leicestershire UK; ^3^ Centre for Crop Systems Analysis Wageningen University & Research Wageningen The Netherlands

**Keywords:** ideotype, root, soil impedance

## Abstract

Mechanical impedance constrains root growth in most soils. Crop cultivation changed the impedance characteristics of native soils, through topsoil erosion, loss of organic matter, disruption of soil structure and loss of biopores. Increasing adoption of Conservation Agriculture in high‐input agroecosystems is returning cultivated soils to the soil impedance characteristics of native soils, but in the low‐input agroecosystems characteristic of developing nations, ongoing soil degradation is generating more challenging environments for root growth. We propose that root phenotypes have evolved to adapt to the altered impedance characteristics of cultivated soil during crop domestication. The diverging trajectories of soils under Conservation Agriculture and low‐input agroecosystems have implications for strategies to develop crops to meet global needs under climate change. We present several root ideotypes as breeding targets under the impedance regimes of both high‐input and low‐input agroecosystems, as well as a set of root phenotypes that should be useful in both scenarios. We argue that a ‘whole plant in whole soil’ perspective will be useful in guiding the development of future crops for future soils.

## SUMMARY STATEMENT

Root phenotypes can be selected to optimize crop performance in response to mechanical impedance in agricultural soils.

## 1 INTRODUCTION

### 1.1 More efficient, resilient crops are urgently needed in global agriculture

In high‐input agroecosystems, intensive use of fertilizers, pesticides and irrigation causes large‐scale environmental pollution and unsustainable resource depletion (Foley et al., 2011; Woods et al., 2010). In low‐input agroecosystems characteristic of developing nations, suboptimal availability of water and nutrients, compounded by biotic stress, are primary limitations to crop production, and therefore, food security, economic development and political stability (FAO, 2015; Nkonya et al., 2016; World Bank, 2017). These constraints are intensifying over time because of the interlinked effects of increasing population pressure, global climate change and soil degradation (Godfray et al., 2010; Mbow et al., 2019; Tebaldi & Lobell, 2008; Tilman et al., 2011). The development of crops with more effective and resilient root systems is a promising avenue to address these challenges. Roots play a central role in crop adaptation to water and nutrient stress, as well as resistance to soil pathogens and pests. Roots, and more generally, the plant–soil interface, including associated microbiota, could also play a key role in ameliorating soil degradation and climate change by improving soil quality and sequestering atmospheric carbon into soil organic matter. To realize this potential, we need to understand the fitness landscape of root phenotypes—how root phenotypes influence soil resource capture and plant performance in various environments—so that root function may be optimized through management and breeding, and in turn, how the future soil environments developed under contemporary agricultural systems will impact the behaviour of different root phenotypes. The scope and diversity of edaphic stresses and plant responses considered require that our focus be on general trends and patterns, for which specific exceptions may exist.

### 1.2 Plant adaptations to degraded soil

FAO estimates that while only about 11% of the earth's surface is devoted to agriculture, approximately 33% of agricultural soils are degraded and over 90% could be degraded by 2050 (FAO, 2011; Montanarella et al., 2018). Compaction is a physical form of soil degradation where soil structure is broken down and pore space is reduced, usually as a consequence of tillage, cropping and grazing (Hamza & Anderson, 2005). In addition to reducing soil capacity for water infiltration, aeration and storage of water and nutrients, the diminished pore space in compacted soils also augments soil strength, thereby mechanically impeding the penetration of roots into the soil matrix. In this article, we focus on how root adaptations to mechanical impedance may have evolved over time, and are continuing to evolve, in response to changing soil environments. We adopt a ‘whole organism in whole soil’ approach to consider how ancestral adaptations to native soil may have changed in response to crop cultivation. We propose that ongoing changes in the soil environments of both high‐input and low‐input agroecosystems may present divergent physical constraints for soil exploration by roots. Finally, we suggest new ideas regarding how the evolving fitness landscape for root phenotypes offers opportunities to breed more resilient, efficient crops. In addition to being more productive, such crops would improve soil quality through biopore development, organic matter accumulation and erosion mitigation. We will not focus on a comprehensive review of all relevant literature, but rather seek to provide new thinking that may catalyse research to address significant knowledge gaps.

## 2 TRAJECTORY OF SOIL IMPEDANCE AS A PRIMARY CONSTRAINT TO ROOT EXPLORATION

In this section, we introduce soil characteristics in key agroecosystems, which are central to understanding how root phenotypes may affect crop performance in those systems.

### 2.1 Soil compaction and impedance to root growth

Compacted soils inhibit root growth through reduced pore connectivity and available space to accommodate the displacement of soil particles as the root apex moves through the soil matrix (Batey, 2009; Hernandez‐Ramirez et al., 2014, Suzuki et al., 2013; Valentine et al., 2012). Soil texture, structure, water content and bulk density are the primary components influencing the penetration resistance of compacted soils to emerging roots. In a growing root, the root cap is advanced through the soil matrix by turgor pressure within the root elongation zone, and if this pressure is not sufficient to overcome resistance at the soil–root interface, elongation of the root will be impeded. Generally, mechanical impedance >2 MPa is sufficient to inhibit the emergence and elongation of roots in both monocot and dicot species (Atwell, 1993; A. G. Bengough et al., 2006; Coelho et al., 2013; Colombi & Walter, 2016; M. T. Grzesiak et al., 2014; S. Grzesiak et al., 2013; Pfeifer et al., 2014). Mechanical impedance of root elongation limits crop productivity by inhibiting soil exploration and soil resource capture, and consequently, is a key factor affecting the energetic cost of soil resource extraction by roots. Beyond directly impeding root emergence and elongation, conditions that increase soil strength also affect soil hydraulic conductivity, water storage capacity, gas diffusivity and permeability, nutrient cycling and the habitat of soil organisms (Keller et al., 2019; Tracy et al., 2011).

### 2.2 Mechanical impedance in native soils

Root systems under undisturbed conditions can proliferate extensively and grow deep into the soil profile. Dickinson and Polwart (1982) recorded a root biomass of around 5 tonnes/ha in the top 15 cm of UK grassland soil. In natural environments, such as where the soil is not subject to regular cultivations and other agri‐management interventions, for example permanent pasture, mechanical impedance to root growth commonly occurs under situations of water deficit, where the drying of shallow soil horizons leads to increased penetration resistance in superficial domains. Previous work on root penetration resistance in the epipedon (i.e., the upper soil layer) has reported a feedback interaction between soil strength, water extraction and root growth. Higher penetration resistance in the topsoil serves to initially limit root system depth, thereby exacerbating topsoil dying, further increasing penetration resistance of the topsoil and decreasing overall depth of the root system (Colombi et al., 2018). The ability of roots to generate the required growth pressure is impaired in drying soils while the strength of the soil increases, thereby increasing the growth pressure required for elongation (Whalley et al., 1998). Reduced pore space also diminishes water storage capacity, so compacted soils dry more quickly than uncompacted soils, further increasing penetration resistance to root growth (Colombi et al., 2018; Lipiec & Hatano, 2003). Decreased hydraulic conductivity in compacted soils also leads to diminished transpiration‐driven mass flow of nutrients to roots (Lipiec & Hatano, 2003; Richard et al., 2001; Romero et al., 2011).

Under native soils, the impacts of grazing (animal trampling) can have significant negative impacts on soil physical properties (Zhang et al., 2019). The extent of soil compaction associated with this depends upon animal type and associated characteristics (e.g., hoof size, animal mass); however, further impacts can be compensated through careful management for example limits to grazing periods, regular relocation of feed/water stations (Taboada et al., 2011). A further limitation to root penetration in natural ecosystems is the increased hardness and density that is commonly observed with increasing soil depth, caused by overburden pressure, effectively the pressure imposed on soil at depth by the weight of the horizons on top of it (Baker et al., 2015). Values >2 MPa (which would impair root elongation) can be readily reached at depths beyond 40 cm, and in some cases, at shallower depths (Gregory et al., 2007). Additionally, some soils naturally have harder subsoils or horizons depending on the soil type and the dominant factors of formation. For example, duplex soils are characterized by horizons with contrasting soil texture, and the sodic subsoil horizons found in South Australia can have a soil strength that significantly restricts root growth (Chittleborough, 1992).

In soils with high bulk density, roots will often forage for zones of weakness in the soil matrix to facilitate easier penetration (Atkinson et al., 2020). In this context, the role of the soil biopores is crucial in assisting with root elongation and proliferation at depth. Biopores are created by soil faunal or root activity and can significantly improve the ability of roots to extend deeper into the soil profile, where high bulk density would otherwise preclude root elongation (Zhou et al., 2021). Hirth et al. (2005) found that soil strength and biopore angle equally impacted the elongation of ryegrass roots, with roots more likely to exit inclined biopores rather than those horizontally orientated. The nature of the soil structure, particularly the shape and size of aggregates, can have important consequences for root development. For example, in tilled soils, aggregates in the seedbed are usually prepared to conform to a granular or crumb‐like structure. These aggregates, often less than a few centimetres in diameter, are often rounded, and when packed together, they generate a structure that is porous, supporting germination, seedling establishment and root elongation (Blunk et al., 2021). However, in native or undisturbed soils, aggregates tend to be larger and more irregular in shape, depending on the dominant factors of soil formation. In subsoil horizons, zones of weakness can exist at the interfaces of packed aggregates, which roots can exploit for easier elongation than in the bulk zone. In this case, the nature of the soil structure can exert a significant influence on the developing root system architecture. For example, roots can extend more readily vertically at depth in a soil with a prismatic or columnar structure that are characterized by narrow horizontal axes and extended vertical axes, which in turn aid the development and stability of the soil aggregates and structure.

### 2.3 Mechanical impedance in cultivated soils

The advent of agriculture and soil tillage was associated with altered soil mechanical impedance. Despite the direct loosening effects of topsoil tillage, such tillage also causes (1) greater topsoil erosion and therefore loss of less consolidated soil horizons, (2) soil hardening via compaction caused by vehicle and animal traffic, (3) reduced soil macrofaunal activity leading to reduced biopore development and (4) greater organic matter oxidation, thereby degrading soil structure and increasing soil bulk density.

#### 2.3.1 Topsoil erosion

The conversion of native vegetation into agriculture is associated with higher rates of soil erosion because of soil disturbance and reduced vegetation cover. This process and its role in the decline of world civilizations have been recognized since the time of Plato (Montgomery, 2007a). Loss of soil from conventionally tilled agricultural fields is 1–2 orders of magnitude faster than from natural landscapes (Montgomery, 2007b). This rate of soil loss exceeds the rate of soil formation by more than a factor of 10 (Montgomery, 2007b). Soil erosion increases the mechanical impedance of the root zone by reducing the thickness of the epipedon, which forces the root zone into subsoil horizons, which often have greater clay content and bulk density, less organic matter content and porosity, and less favourable nutrient‐ and water‐holding characteristics. Conservation Agriculture practices (i.e., using residues as a soil amendment and protector of the soil surface and intercropping/cover cropping as part of a rotation, along with efforts to minimize soil disturbance) are associated with greatly reduced rates of soil erosion that are comparable to natural soil formation rates (Montgomery, 2007b), and are therefore a key component of sustainable agriculture (see Section 2.4). Nonetheless, historically, soil erosion in agricultural landscapes has created a more challenging environment for root growth in the context of mechanical impedance.

#### 2.3.2 Reduced soil organic matter

**Figure 1:**
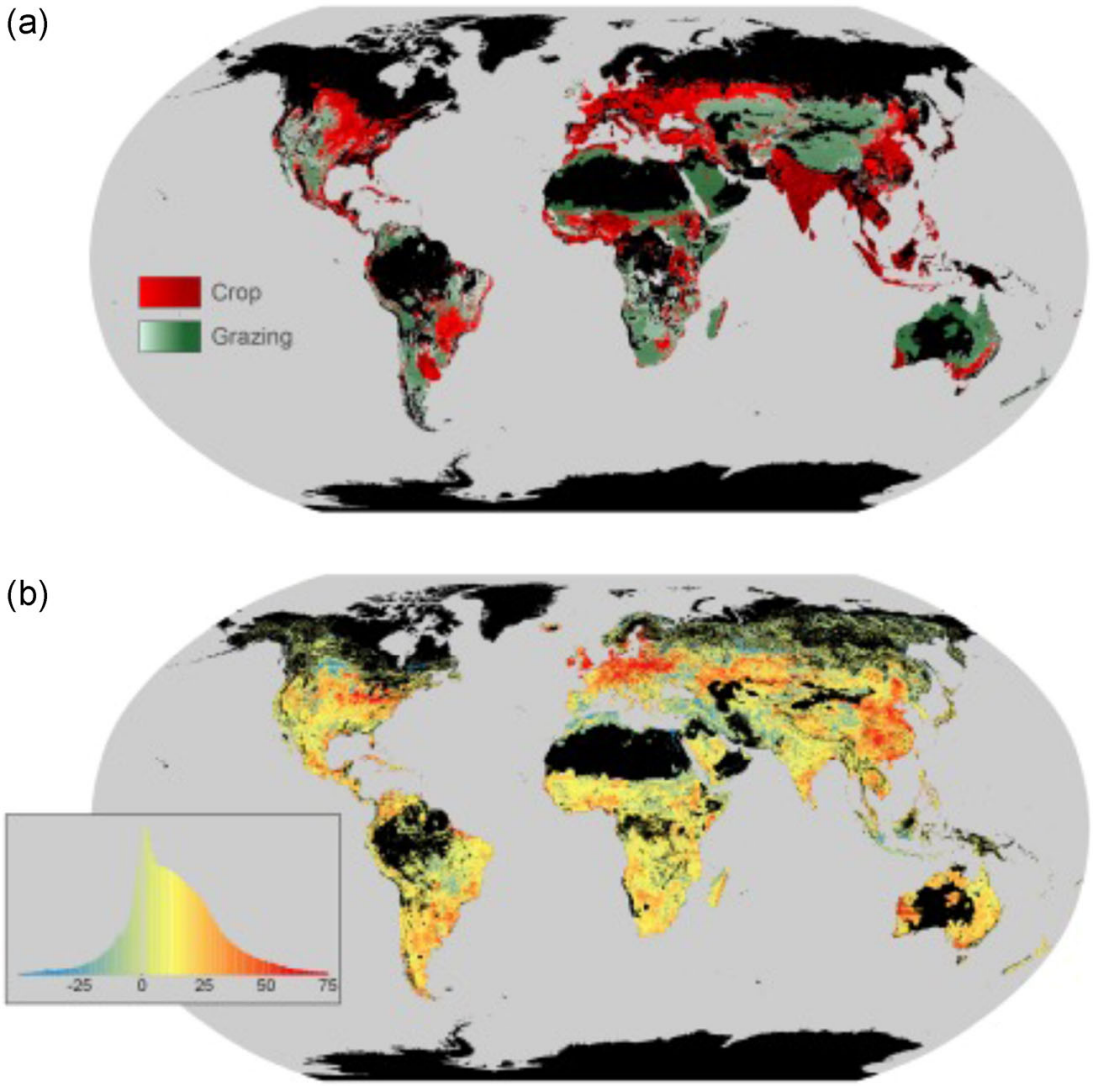
Global distribution of cropping and grazing in 2010 from (a) HYDE v3.2 and (b) modelled SOC (soil organic carbon) change in the top 2 m. In (a), colour gradients indicate the proportion of grid cell occupied by given land use. In (b), the legend is presented as a histogram of SOC loss (Mg C per hectare), with positive values indicating loss and negative values depicting net gains in SOC. From Sanderman et al. (2017) [Color figure can be viewed at wileyonlinelibrary.com]

Of all the impacts on soil from modern agriculture, one might argue that the key concern has been the systematic degradation of soil carbon stocks associated with extensive agricultural mechanization, and in particular, the action of tillage, due to its importance in sustaining soil productivity. It has been estimated that 133 billion tonnes of carbon, or 8% of the total global soil carbon stock, may have been lost from the upper 2 m of the world's soil since the start of the first agriculture, known as the total ‘soil carbon debt’ (Sanderman et al., 2017; Figure 1). The largest losses have been linked to arable crop production in the US Corn Belt and Western Europe since the start of the industrial revolution. In the United States, as much as 30%–50% of soil organic carbon that the soil contained before the establishment of agricultural production system has been lost (Kucharik et al., 2001). Similarly, Bellamy et al. (2005) reported an annual loss of 0.6% (relative to the existing soil carbon content) between 1978 and 2003 for England and Wales (Bellamy et al., 2005). Sanderman et al. (2017) found median losses of soil organic carbon of 26% from the upper 30 cm following a meta‐analysis, albeit with a range of −36% to 78%.

#### 2.3.3 Soil compaction

Soil compaction due to vehicle traffic is an issue affecting an estimated 68 million ha worldwide (Hamza & Anderson, 2005), a number that has likely increased in recent years as the mechanization of agriculture becomes more widespread and the size of agricultural machinery increases (Keller et al., 2019; Schjønning et al., 2015; Tracy et al., 2011). In contrast to visually obvious forms of soil degradation like erosion and salinization, compaction can be difficult to diagnose in the field without specialized equipment such as a penetrometer. In sandy soils, subsoil compaction may not significantly interfere with water infiltration into deep layers, but can prevent roots from reaching and utilizing this water, leading to poor water use efficiency and increased drought hazard (Laker, 2001). In intensively managed agricultural systems, issues of compaction are often exacerbated in a positive feedback cycle, where increased soil strength and decreased soil fertility reduce plant growth, leading to lower inputs of crop residues, thereby reducing nutrient cycling and mineralization of carbon and nitrogen as well as activity of micro‐organisms (De Neve & Hofman, 2000). Specifically, soil organic matter affects soil structure and compactability through binding of soil particles, reducing the wettability and increasing the mechanical strength of soil aggregates (Tisdall & Oades, 1982). Ultimately, soil texture strongly influences the capacity for soil to accumulate C, where clay particles form recalcitrant clay–humic complexes with low recycling rates due to their size, electrical charge and high surface area to volume ratio (Six et al., 2002). Additionally, the ratio of C:N of organic matter can affect the rate of decomposition by bacteria. Crop residues that have a low C:N ratio and high soluble carbohydrate content will ultimately enhance CO_2_ emissions and lead to the further depletion of soil organic carbon stocks (Novelli et al., 2011). Compaction of agricultural soils has also been shown to reduce the concentration of CO_2_ held within the soil (Conlin & Driessche, 2000).

#### 2.3.4 Reduced biopore development

**Figure 2:**
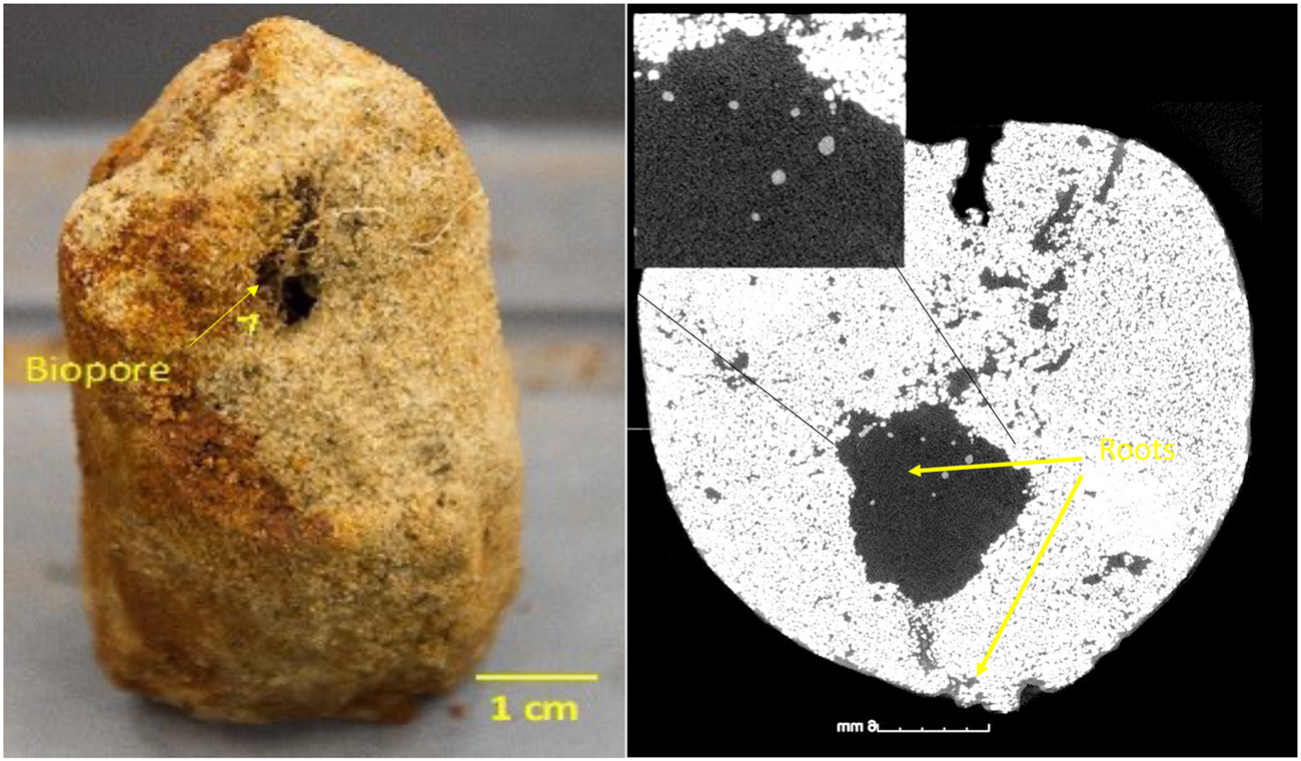
Example of biopore development in a sandy loam soil from 60 to 70 cm soil depth with roots clearly visible entering a biopore (left) and shown clustered in a biopore (right) from an X‐ray computed tomography image taken in cross section (*x*–*y* plane; inset shows roots at higher magnification). Resolution = 20 µm. Adapted from Zhou et al. (2021) [Color figure can be viewed at wileyonlinelibrary.com]

Soil biopores are stable networks of channels produced by roots, earthworms and macrofauna (Kautz, 2014). Several studies report preferential root growth in biopores, especially in hard soils (Cresswell & Kirkegaard, 1995; Landl et al., 2019; Watt et al., 2006; Figure 2). Biopore networks in the topsoil can be disrupted by conventional tillage and vehicle traffic (Dexter, 1979; Hadas, 1997), resulting in reduced biopore frequency in tilled agricultural soils (Or et al., 2021, Figure 3). Even in tilled soils, biopore networks may persist in the subsoil, which could benefit resource capture by deep roots (Kirkegaard & Lilley, 2007; Lucas et al., 2019; McCallum et al., 2004; Watt et al., 2006).

**Figure 3:**
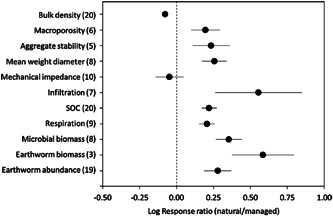
Metrics of natural soil structure relative to managed soil structure. Data are from paired studies that compare metrics of natural and managed soil structure from the same location. Numbers in parentheses indicate the number of paired studies. From Or et al. (2021)

### 2.4 Changing soils associated with modern agriculture

Pressures from a changing climate will bring about complex and systematic changes to soils and impinge on their abilities to provide essential functions (Haygarth & Ritz, 2009). Whilst the value of terms such as soil quality and soil health is currently heavily debated in the literature (Kibblewhite et al., 2008; Powlson, 2020), it is widely accepted that new approaches will be needed to ensure that soils are fit for purpose in the future. Whilst neglect and overcropping can lead to significant loss of soil carbon (see Section 2.3), restoration is not without considerable challenges. It is estimated that a shift towards carbon‐enhancing management practices for example use of fallow periods, effective manure management, and so forth could recover up to two thirds of carbon losses (Smith et al., 2008). However, Sanderman et al. (2017) dispute this, suggesting that even with renewed focus on the sustainable management of soils, the global carbon sink that can be filled is at best 10%–30%.

Conservation of soil as an indispensable resource must be a future priority. Loss of soil via erosion represents a significant global agricultural problem. However, recent suggestions of a short lifespan of global soils (the so‐called ‘60 harvests left’; Wong, 2019) have been discredited (Evans et al., 2020), with only 16% of soils managed by conventional approaches having a lifespan <100 years based on net erosion. Conversely, for soils managed according to the principles for Conservation Agriculture, this value decreases to 7%, which gives a clear steer towards safeguarding future soils; even though the productive lifespan of most soils exceeds several hundred years, the global trend is predominantly towards soil thinning.

In particular, recognition of the effects of intensive cropping and continued tillage has become more widespread and it would appear that a global transition to minimum and no‐till systems has started, albeit with considerable geographical variability. Indeed, the key principles of Conservation Agriculture, that is using residues as a soil amendment and protector of the soil surface and intercropping/cover cropping as part of a rotation, along with efforts to minimize soil disturbance, have received considerable attention particularly in the last 10 years. The decision for a farmer to till or not involves a considerable number of factors including soil/crop type, availability of machinery, cost of fuel, impact on yield, and so forth. It is well recognized that no‐till systems, early after conversion from conventional tillage, tend to have dense topsoil that has greater mechanical impedance for root growth in shallow horizons (Colombi et al., 2018). This also leads to reduced infiltration (Mangalassery et al., 2014) and can lead to negative impacts on yield. However, there is considerable conflicting literature regarding the impact of no tillage on yield (Mangalassery et al., 2015). Over time, under no till conditions, soil fauna can operate and proliferate without disturbance, which helps to generate a new soil structure developed under more natural conditions. It was recently shown in the United Kingdom that after 7 years, uncultivated soils begin to function similarly to cultivated soils hydraulically for example hydraulic conductivity; in addition, there are benefits from improved carbon sequestration and reduced greenhouse gas emissions (Cooper et al., 2021). However, the time scales involved in the development of soil structure, replenished soil organic matter and greater biopore frequency that facilitates the ready development and expansion of crop root systems are likely to vary considerably among different climatic systems and soil types. Thus, it seems likely that our future soils under Conservation Agriculture, especially in the epipedon, are going to endure periods (possibly between 5 and 10 years) of high mechanical impedance until the processes of soil structural development have regenerated soils, many of which have undergone decades of degradation. In this regard, it is vital that the future plant phenotypes that can thrive under such harsh conditions are prioritized.

While global drought predictions suggest a significant increase in frequency (Shukla et al., 2019), some form of mitigation may be offered by no till management, which enhances water retention through an increase in soil bulk density and increased number of micropores (<30 µm). Conversely, under wet conditions, it is likely that anoxic conditions are more likely to develop under no till systems. Under such conditions, the development of soil biopores predominantly via earthworm burrowing or cover crops, preferentially selected due to their deeper rooting (e.g., tillage radish), could be valuable (Burr‐Hersey et al., 2017). After 6–10 years of no till, the total porosity was greater under cultivated soils, while the connected porosity, likely important for infiltration and drainage, was actually greater under no till, which they attributed to the biopores created by earthworms and root channels (Cooper et al., 2021). Management systems, which promote the development of biopores especially at depth in soils, should be prioritized for our future soils. Biopores facilitate deeper root growth, which supports acquisition of water and nutrients. Zhou et al. (2021) recently demonstrated, using X‐ray imaging of undisturbed 1 m long field cores, that below 50 cm depth, wheat roots are predominantly found clustered in biopores and that the soil porosity at depth explained c. 60% of the variance in the root number density (Zhou et al., 2021). This demonstrates the potential benefits for deeper rooting phenotypes and targeting deeper rooting cover crops. In addition, deeper rooting supports greater and deeper carbon additions into the soil, building soil health and resilience. Managing rotation, strategic use of cover crops and intercropping to enhance soil structure to facilitate deeper rooting and building carbon stocks should be key future priorities in attempts to recarbonize soils and support regeneration of soils following decades of neglect.

### 2.5 Soil degradation in low‐input agricultural systems

Low‐input agriculture is a dominant agroecosystem in many developing nations of the tropics and subtropics, characterized by smallholder farmers with limited access to purchased inputs. Mollisols and Alfisols, derived from native prairies and forests, are considered the most agriculturally productive soils, but only make up around 17% of global soils (Eswaran et al., 2012). In contrast, approximately 40% of global landmass is located in the tropics and subtropics, where some of the most common soil orders include Entisols, Ultisols and Oxisols. In general, these soils are highly weathered and weakly structured, with low water‐holding capacity, cation exchange capacity and nutrient content (Bekunda et al., 2010). These soils are often infertile, having low pH and poor bioavailability of key nutrients including P, K, Ca and Mg, in addition to toxic levels of Al and Mn (Lynch, 2019; Sanchez, 2019). Additionally, the high rate of decomposition in these climates severely decreases the organic matter content of tropical soils. For example, soils sampled throughout sub‐Saharan Africa have been reported to have 40% lower carbon stocks compared to the global average (Mokwunye et al., 1996; Smaling & Dixon, 2006).

While potential yield losses due to soil degradation may be masked in high‐input systems through the use of irrigation, fertilizer and technologies like subsoil tillage, the productivity of low‐input agricultural systems throughout the developing world is overtly impacted by poor soil quality. While smallholder farmers are not compacting soils with heavy machinery, these low‐input systems ultimately require more frequent tillage of the topsoil in the form of mechanical weed control (Flach, 1990). Consequently, erosion from continuous tillage has been shown to cause yield reductions of 30%–90% in some shallow soils found in west Africa (Lal, 1987; Mbagwu et al., 1984). Topsoil erosion is especially harmful to crops in weathered soils, since the subsoil is a harsh environment for root growth, being more acidic, with less nutrient availability, and greater Al toxicity (Lynch & Wojciechowski, 2015). Where smallholder farmers lack equipment for subsoiling, continuous tillage of shallow soil horizons and livestock traffic may cause compaction to build up in deeper horizons over time, thereby limiting the depth of the root system. Because the use of irrigation is also limited in low‐input systems, a shallow distribution of root length in the soil profile results in an increased drought hazard (Colombi et al., 2018).

As cultivation techniques used by smallholder farmers are generally driven more by subsistence rather than commercial production, and land availability is often a limitation, fallowing to allow restoration of soil fertility is less routinely employed, especially as populations are rapidly increasing in these regions. As a result, it is estimated that the annual depletion rates in soil fertility across 38 countries in sub‐Saharan Africa are 22 kg N, 3 kg P and 15 kg K per hectare on average (Stoorvogel et al., 1993). Overall, it is estimated that only 29% of soils across the continent of Africa have medium‐high suitability for agriculture, but in reality, land availability is even less due to a rapidly expanding population and competition with other land uses (Eswaran et al., 1997).

This combination of native attributes of tropical soils coupled with the agronomic practices utilized by smallholder farmers portends increasing challenges for agricultural productivity of low‐input systems in the future. The low capacity for biomass production and a high rate of decomposition will serve to further reduce the organic matter content of tropical soils over time. Frequent tillage of shallow soil horizons for weed control will contribute to continued loss of P and N, as well as the deterioration of aggregates and soil structure, thereby further reducing fertility and water‐holding capacity. The lack of subsoil tillage will inhibit root elongation into deeper soil horizons, which will have significant implications for the productivity of these rainfed systems as climate change progresses and the duration and frequency of drought events increase.

## 3 ROOT ADAPTATIONS TO IMPEDANCE

While the soil physical properties of both high‐ and low‐input agricultural systems pose distinct challenges to the crops that they support, the development of novel varieties with optimized root systems can play a key role in improving yields in degraded soils. Ultimately, the integration of soil management strategies coupled with breeding efforts for targeted root architectural and anatomical phenotypes will not only help to ameliorate stress‐induced yield loss in soils with high mechanically impedance to root growth but can also limit further soil degradation. Genetic variation for root architectural and anatomical phenotypes can have dramatic effects on the ability of roots to penetrate strong soils. Root architectural phenes influence the spatial and temporal placement of roots in the soil matrix, and anatomical phenes affect the metabolic costs and influence root penetration in soils with strong mechanical impedance to root growth. Here, we highlight several root adaptations that influence root penetration in hard soils that we believe merit further investigation.

### 3.1 Root anatomical phenes influence the metabolic costs of root tissue

Root phenotypes that reduce the metabolic cost of root production and maintenance benefit plant performance by expanding the volume of soil explored per unit of plant internal resource invested, in addition to release of internal resources for other plant functions such as the growth of photosynthetic tissue and reproduction (Lynch, 2015). Root anatomical phenes are a primary determinant of the metabolic costs of root construction and maintenance, and therefore, the cost of soil exploration (Lynch et al., 2021). The metabolic cost of roots is dependent on the type and proportion of specific tissues (e.g., cell wall, cytoplasm and vacuole) as different tissues have different construction and maintenance costs. Additionally, many anatomical features also influence the proportion of living and dead cells in root tissue, which has a significant influence on the metabolic cost of soil exploration. For example, the formation of root cortical aerenchyma (J. G. Chimungu, Maliro, et al., 2015; Galindo‐Castañeda et al., 2018; Saengwilai et al., 2014), root cortical senescence (Schneider, Postma, et al., 2017; Schneider, Wojciechowski, et al., 2017), increased cortical cell size (Chimungu et al., 2014b), reduced cortical cell file number (Chimungu et al., 2014a) and reduced secondary growth (Strock et al., 2018) have utility in edaphic stress by reducing the metabolic cost of root construction and maintenance. Under circumstances of high soil strength, roots have greater metabolic costs for root elongation, due to root thickening (Vanhees et al., 2020; Yang et al., 2019). However, wheat genotypes with greater cortical cell diameter significantly reduced the energy costs of root growth in soils with greater mechanical impedance (Colombi et al., 2019). In addition, root exudates and mucilage, influenced by species, tissue age and environmental factors, also represent significant carbon costs (Marschner, 1995). Young plants can exude 30%–40% of their fixed carbon as root exudates (Whipps, 1990). Anatomical features that reduce the metabolic costs of root construction and maintenance and optimal root exudation may be beneficial in environments with hard soils by releasing internal resources that can be allocated to further root growth or reproductive tissues. For example, to avoid soil hardening due to receding water tables and soil drying from the surface, a strategy for annual plants would be to grow deep roots quickly, to allow continued growth of the root tip in moist and favourable soil conditions. Anatomical phenes that enable deep rooting at a reduced metabolic cost may be adaptive in these environments (Lynch et al., 2021).

### 3.2 Root phenotypes are important for penetrating hard soils

**Figure 4:**
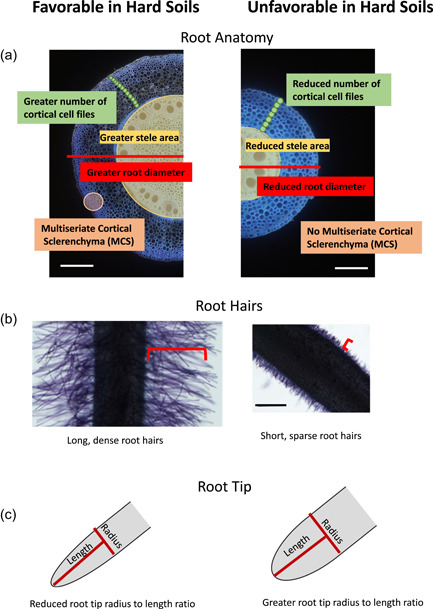
Root phenes for enhanced root penetration in hard soils. (a) Roots with a greater root diameter, greater stele area, many cortical cell files, small cortical cells in the outer cortex, (b) long, dense root hairs and (c) and a reduced root tip radius to length ratio enhance root penetration in hard soils [Color figure can be viewed at wileyonlinelibrary.com]

Intraspecific variation in root penetration ability in several crops including maize (Bushamuka & Zobel, 1998; Chimungu, Loades, et al., 2015; Vanhees et al., 2020), lupin (Chen et al., 2014), wheat (Botwright et al., 2007; Kubo et al., 2006) and common bean (Rivera et al., 2019) suggest the adaptive value of root traits for enhanced penetration in hard soils (Figure 4). Several root phenotypes improve penetration of hard soils. In wheat, roots with a steep growth angle are associated with enhanced root penetration of hard soils (Vanhees, Schneider, et al., 2021; Whalley et al., 2013). Anatomical phenes are also important for enhanced penetration ability. In maize grown in strong soils, deeper rooting was associated with greater cortical cell file number and greater mid‐cortical cell area at node three and greater root cortical aerenchyma formation at node four (Vanhees et al., 2020; Figure 4a). Small outer cortical cells aid in stabilizing the root against ovalization and reduce the likelihood of buckling. Cortical cell thickness, cortical cell count, cortical cell wall area, outer cortical cell size and stele diameter are all associated with increased root penetration and bend strength (Chimungu, Loades, et al., 2015). In hard soils, maize roots become coarser (Vanhees, Loades, et al., 2021) and wheat roots have a plastic response in the expression of root cross‐sectional area, stele and cortical area, root cortical aerenchyma and cortical cell size and file number (Colombi & Walter, 2017). Multiseriate cortical sclerenchyma (MCS) is characterized by small cells in the outer cortex with thick, lignified cell walls (Schneider et al., 2021). In maize and wheat, MCS increases root tensile strength and therefore increases rooting depth in strong soils. In maize, genotypes with MCS have greater rooting depth and greater shoot biomass in compacted soils in the field (Schneider et al., 2021).

Increased root diameter may also facilitate root anchorage, thereby improving penetration of hard soils. Root anchorage, through the production of root hairs and lateral roots and the friction of soil particles, may support the maximum growth pressure (i.e., growth force exerted by the root per unit cross‐sectional area), which is largely driven by turgor pressure in the expanding cells at the root elongation zone, to force the root tip to continue root elongation into hard soils (A. Bengough & Mullins, 1990; Lynch & Wojciechowski, 2015; Figure 4b). Radial thickening may also relieve stress from the root tip by deforming the soil near the root tip through an increase in the number of cell files, cell size or cell wall thickness (Atwell, 1993).

### 3.3 Root thickening in response to impedance may be a ‘stop signal’ for root growth

Generally, roots with greater diameter have been considered superior in penetrating strong soils. Thick roots are more resistant to buckling and deflection upon encountering soils with greater mechanical impedance (Whiteley et al., 1982), and root thickening is a common response to hard soils (Araki et al., 2000; Atwell, 1993; Colombi & Walter, 2016). In response to compaction, increases in root diameter were due to the addition of cell layers radially (Colombi et al., 2019; Vanhees et al., 2020) and the expansion of cortical cells (Atwell, 1989; Colombi & Walter, 2017; Vanhees et al., 2020; Vanhees, Schneider, et al., 2021). However, an increase in root diameter in response to hard soils may also be accompanied by a decrease in cell flux and division at the apical meristem (Clark et al., 2003; Croser et al., 1999) and therefore root elongation (Atwell, 1993). Therefore, roots of augmented diameter in hard soils have decreased root elongation and root depth (Colombi et al., 2019; Vanhees et al., 2020; Vanhees, Schneider, et al., 2021). Upon encountering hard soils, the length of the elongation zone is reduced in response to decreased cell wall extensibility in the axial direction at the apical end of the elongation zone, while the local growth rate is maintained (Bengough et al., 2006). The abbreviated elongation zone will therefore produce cells with a smaller length and larger diameter (Bengough et al., 2006; Colombi et al., 2019). Elongation rates are related to the length of the elongation zone and therefore by increasing the length of the elongation zone and the cell wall properties of the expanding cells, mechanically impeded roots may be able to continue to elongate rapidly (Bengough et al., 2006; Sharp et al., 2004).

Greater root diameter is associated with greater metabolic cost of root elongation (Colombi et al., 2019; Vanhees et al., 2020; Yang et al., 2019). In a recent study, root thickening was not related to deeper rooting in compacted soils; however, root anatomical phenotypes influenced rooting depth, especially in thinner roots of older nodes in maize (Vanhees et al., 2020). Ethylene may be an important signal in response to strong soils by causing radial thickening and slowing root elongation (Vanhees, Schneider, et al., 2021). This ‘stop’ signal may be adaptive by redirecting soil foraging by the root system as a whole to softer, wetter soil domains. Ethylene diffusion is reduced in compacted soils and therefore accumulates near root tissues, which triggers a signalling cascade to stop root growth (Pandey et al., 2021). In maize and other cereals, thinner roots may have a greater propensity to thicken when compared to younger, thicker roots emerging at more mature growth stages. Innately thicker roots may experience less impedance stress than thinner roots and, therefore, young, thick roots that do not respond to hard soils by radially expanding may be beneficial for growing though strong soils. Root thickening is node‐specific, influenced by several root anatomical phenes, and may be obscured by allometric effects (i.e., effects driven by plant growth and size, Vanhees et al., 2020). The utility of roots that are innately thicker compared to roots that thicken as a response to strong soils remains to be explored.

### 3.4 Root tip geometry and mucilage are important for root penetration

Root tip geometry also plays an important role in influencing root elongation and penetration in strong soils (Figure 4c). For a root to penetrate soil, it must overcome the frictional resistance between the root and soil and requires pressure to expand the cavity in the soil. The geometry of the root tip, rate of penetration and soil mechanical properties determine the cavity expansion pressure required for penetration. A smaller root tip radius to length ratio reduces penetration stress and enables root elongation in hard soils (Colombi et al., 2017). More narrowly pointed root tips have more efficient, cylindrical‐like deformation of the soil when compared to less efficient spherical‐like deformation of the soil caused by more blunt root tips (A. G. Bengough et al., 1997). In addition, mucilage or rhizo‐deposits may alter the mechanical properties around the root tip including hydraulic properties of the rhizosphere (Carminati et al., 2010; Read et al., 2003; Whalley et al., 2005). Roots produce more border cells and mucilage in mechanically impeded soils (Barber & Gunn, 1974; Iijima et al., 2000). A better understanding is required of how root mucilage and exudates interact with the soil at the root tip to influence penetration in hard soils.

## 4 THE FITNESS LANDSCAPE FOR ROOT ADAPTATIONS TO IMPEDANCE MAY HAVE CHANGED

**Figure 5:**
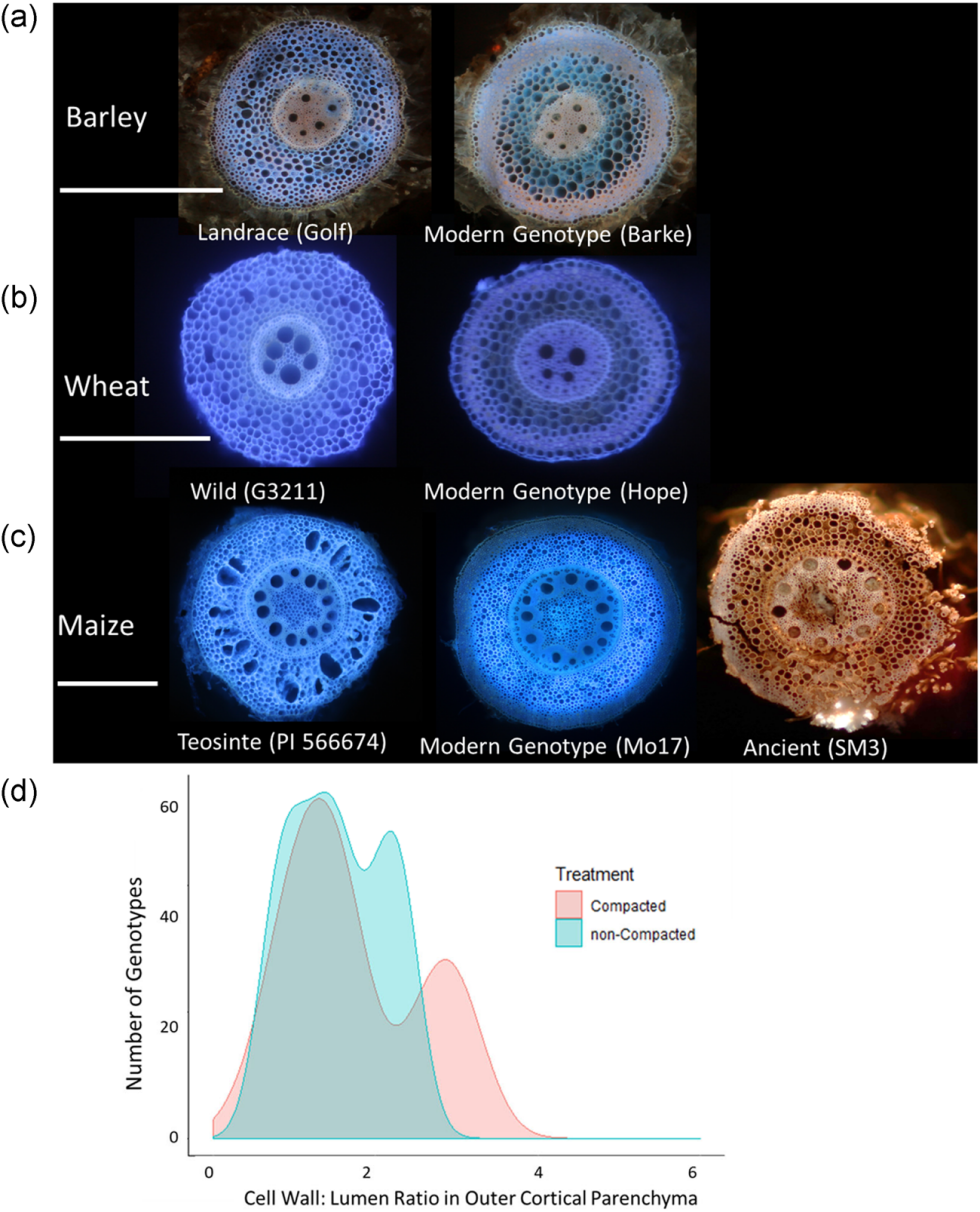
Modern genotypes may develop multiseriate cortical sclerenchyma (MCS), but not landraces or wild ancestors. MCS is characterized by small cells with thick cell walls in the outer cortex. (a) MCS is not observed in barley landraces, but is observed in approximately 60% of modern genotypes. (b) MCS is not observed in wild wheat ancestors, but is observed in approximately 40% of modern genotypes. (c) MCS is not observed in teosinte, but is observed in approximately 30% of modern maize inbred genotypes. MCS was observed in an ancient maize root specimen dating 5280–4970 before present (BP). (d) MCS is plastic in compaction stress. Maize inbred lines that were grown in compacted soils developed thicker cell walls in MCS after growing through a compacted layer. Images modified from Schneider et al. (2021) and López Valdivia et al. (2019). Data from Schneider et al. (2021). Scale bars = 500 µm [Color figure can be viewed at wileyonlinelibrary.com]

Recent evidence supports the hypothesis that the fitness landscape for root adaptations to mechanical impedance has changed through crop evolution from wild ancestors. MCS, characterized by thick, lignified cell walls in the root cortex, is heritable, genetically controlled and shows variation in many Poaceae species including maize, wheat, barley and sorghum. A recent study evaluating maize, wheat and barley wild crop ancestors, landraces and modern cultivars found that wild crop ancestors and landraces did not develop MCS, while MCS was present in many modern cultivars (Figure 5). In addition, MCS is modulated by ethylene, an important signal in compaction stress. Genotypes that constitutively do not form MCS developed MCS upon exogeneous ethylene exposure (Schneider et al., 2021). Presumably, ethylene signals play a role in MCS plasticity upon compaction stress. Plants show plasticity in the degree of MCS expression as cortical cell wall thickness is modified as the root grows into less compacted from more compacted soil layers (Schneider et al., 2021). We propose that MCS is an adaptive trait for soil resource acquisition in modern agroecosystems. The responsiveness of MCS to ethylene, its plastic response to greater mechanical impedance and its utility in penetrating hard soils may be useful for penetrating hard soils caused by agricultural tillage and especially mechanization (see Section 5). The presence of MCS in cultivated taxa but its apparent absence from wild crop relatives supports the proposal that agriculture has changed the fitness landscape of roots in terms of mechanical impedance.

Presently, literature focused on root growth in soils with high penetration resistance places emphasis on the identification of root adaptations for improved exploration into strong soils (Colombi & Keller, 2019; Lynch & Wojciechowski, 2015). Alternatively, little attention has been paid to the inverse of this approach, where the economic framework of root system development may indicate that the inhibition of root elongation into domains with high impedance is an adaptive response rather than a symptom of limited growth capacity. Here, we propose that active suppression of root elongation into strong soils would likely benefit the plant if the energetic investment of exploring impeded domains surpasses the payoff in resource acquisition. By restricting root growth into strong soils, plants may then reallocate internal resources to root growth into soil domains where this balance between resource expenditure and acquisition is improved. For the ultimate goal of improving crop performance in conditions of greater mechanical impedance, understanding of the costs and benefits of root penetration under different distributions of soil strength at a whole‐plant level is essential.

**Figure 6:**
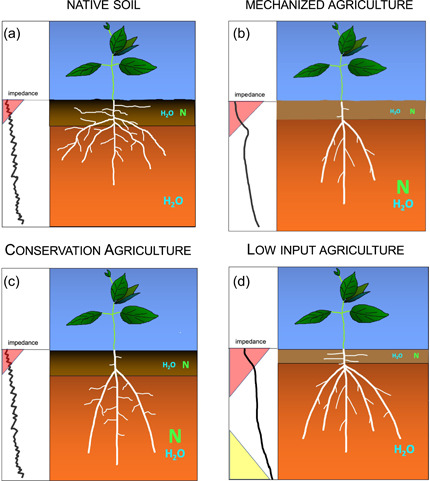
Conceptual scheme of four soil scenarios, their impedance profiles (shown in the left portion of each panel as increasing impedance from left to right) and hypothetical root phenotypes adapted to them, as described in the text. (a) Native soil: Mechanical impedance to root growth in native soils is mediated by high organic matter content, low‐resistance pathways formed by biopores, soil aggregates and soil structure and drought‐induced hardening of the topsoil (pink triangle), with N and water available in the topsoil, but greater water availability at depth. Nitrogen availability is limited and is greater in the epipedon from organic matter mineralization. We propose that root phenotypes adapted to this environment have plastic roots that can respond to local low‐resistance pathways, and will benefit from dimorphic root phenotypes that promote both topsoil and subsoil foraging. (b) Soils under conventional tillage, which, in comparison to native soil, have a thinner epipedon with less organic matter, hence less water‐holding capacity and greater susceptibility to soil hardening due to soil drying, fewer low‐resistance pathways from soil structure and biopores and often have a plowpan from vehicle traffic. Over time, nitrogen availability is greater at depth due to nitrate leaching from fertilizer. In these environments, nonplastic root phenotypes that can penetrate through hard surface layers to reach deep soil domains with greater water and N availability could be advantageous. Root phenotypes that promote topsoil foraging could be less useful for mature plants. (c) Conservation Agriculture. In high‐input agroecologies, traditional tillage in mechanized agriculture is evolving towards reduced tillage in Conservation Agriculture, which will return to some of the features of native soil, including greater topsoil organic matter, greater frequency of biopores, greater aggregate development and improved soil structure, but harder bulk soil, and greater N availability in deep strata because of nitrate leaching from fertilizer. More plastic root phenotypes that avoid hard, dry soil domains to exploit biopores, soil fissures and deeper, wetter and therefore softer soils could be advantageous. Penetrating axial roots, parsimonious root phenotypes and phenotypes that support subsoil exploration could be useful in exploiting N and water in deep soil strata. (d) Soils under low‐input agriculture, with similar characteristics as mechanized agriculture, but with greater loss of the epipedon and organic matter, hence greater susceptibility to soil hardening due to soil drying, no plowpan, low N availability limited to the epipedon because of limited fertilizer use and the additional barrier of acid subsoil (yellow triangle). In these environments, nonplastic root phenotypes that can penetrate through hard surface layers to reach deep soil domains with greater water availability will be advantageous, along with Al tolerance and dimorphic root phenotypes that also permit capture of shallow N from mineralization [Color figure can be viewed at wileyonlinelibrary.com]

We propose that the evolution of soil environments can be grouped into four categories: (1) native soil, (2) traditional mechanized agriculture, (3) Conservation Agriculture and (4) low‐input agriculture (Figure 6).

*Native soil*: Mechanical impedance to root growth in native soils is mediated by high organic matter content, low‐resistance pathways formed by biopores, soil aggregates and soil structure and drought‐induced hardening of the topsoil, with N and water available in the topsoil, but greater water availability at depth (Figure 6a).

*Mechanized agriculture*: In contrast to native soils, soils under conventional tillage have less organic matter, thinner topsoil, fewer biopores, less favourable soil structure at several scales, and thus fewer low‐resistance pathways for root growth, less N‐ and water‐holding capacity in the topsoil and greater tendency towards topsoil drying and hardening, but much greater N availability in the subsoil because of leaching of fertilizer N (Figure 6b).

*Conservation Agriculture*: In high‐input agroecosystems, traditional tillage in mechanized agriculture is evolving towards reduced tillage, which will return to some of the features of native soil, including greater topsoil organic matter, greater frequency of biopores, greater aggregate development and improved soil structure, but harder bulk soil, and greater N availability in deep strata because of nitrate leaching from fertilizer (Figure 6c).

*Low‐input agriculture*: These environments are the most challenging for root growth, with loss of topsoil and organic matter, a high rate of drought‐induced hardening of the topsoil and limited nutrient and often water availability, with the added constraint in weathered soils of acid subsoil (Figure 6d).

## 5 ROOT IDEOTYPES FOR FUTURE SOILS

Ideotypes are conceptual models of ideal phenotypes to be targeted in crop breeding programmes. Ideotype breeding has several advantages over brute‐force yield selection, especially with complex breeding goals such as improved adaptation to edaphic stress (Lynch, 2019). Here, we propose ideotypes for crop adaptation to future soils in the context of mechanical impedance that we believe merit investigation and validation.

### 5.1 Root ideotypes for high‐input systems

The current trajectory for soils in high‐input systems is moving gradually towards the principles of Conservation Agriculture, which will, over time, lead to greater soil organic matter content, improved soil structure through aggregation and increased number of biopores. These trends will be strengthened if carbon markets are developed to compensate farmers for soil carbon sequestration. Climate change is forecast to intensify water deficit stress in many high‐input agroecosystems, and so drought‐induced soil hardening, especially in the topsoil, will be an important constraint to root growth. This will create a scenario in which a soil environment more like that of native soils will confer fitness benefits to plants with more ancestral root phenotypes in terms of mechanical impedance (Figure 6c). More plastic root phenotypes that avoid hard, dry soil domains to exploit biopores, soil fissures and deeper, wetter and therefore softer soils would be advantageous (although soils that are too soft are not desirable either). These traits may be associated with greater ethylene responsiveness. Crops in high‐input systems, in which drought is important but biotic stresses are managed, may also benefit from parsimonious root phenotypes that focus on rooting depth at the expense of other root functions (Lynch, 2018).

### 5.2 Root ideotypes for low‐input systems

Soil degradation, primarily wind and water erosion, is likely to continue to be a dominant factor in the fertility of low‐input systems. Climate change is likely to exacerbate soil degradation by increasing the severity of rainfall events, and by increasing water deficit stress, thereby reducing the protective effects of crop cover and carbon inputs from crop residues. These trends are autocatalytic in that increased soil degradation exacerbates crop water and nutrient stress, which in turn reduces nutrient cycling and carbon inputs, making soils more vulnerable to degradation. These trends will lead to soils with less topsoil, less organic matter, reduced water‐holding capacity and therefore more prone to water deficit, and greater mechanical impedance. In such soil environments, root phenotypes that can penetrate through hard surface layers to reach deep soil domains with greater water availability will be advantageous (Figure 6d). These phenotypes would need to be less plastic in response to soil impedance, and possibly less responsive to ethylene, opting instead to penetrate hard soil to reach deep water. The fact that many such systems are in acid soils with increasing aluminium toxicity and decreasing nutrient availability with soil depth represents an additional challenge (Lynch, 2019). Nitrogen availability is a limitation in these systems and is concentrated in the epipedon from mineralization of soil organic matter, so dimorphic root phenotypes that are capable of subsoil as well as topsoil foraging will be advantageous (Burridge et al., 2020; Lynch, 2019).

### 5.3 Root ideotypes for both high‐input and low‐input systems

Several root phenotypes are likely to be useful across a range of soil environments, including high‐input and low‐input systems. These include the following:

1. *Long, dense root hairs*: Root hairs provide anchorage as roots elongate, aiding in penetration of hard soil, and are critically important for the acquisition of immobile soil resources such as phosphorus, which are critical limitations in low‐input agroecosystems. Crops show substantial interspecific variation for root hair phenotypes, and this variation is easily screened. Crops with long, dense root hairs will be increasingly useful in future soils as we work to make high‐input systems less demanding and more resilient, and low‐input systems more productive (Lynch, 2019).
2. *Cheap roots*: An array of anatomical phenotypes can reduce the metabolic cost of soil exploration, thereby improving water and nutrient capture, and penetration of hard soil (see Section 3.1; Lynch, 2019). These should be useful in an array of soil environments.
3. *Developmental regulation of root plasticity in cereal crops*: In cereal crops, distinct root classes are produced over time. The primary root is followed by seminal roots; then, nodal roots produced by shoot nodes that emerge near or above the soil surface. Distinct root classes may have different plastic responses to impedance, as has been shown for example in wheat (Colombi et al., 2017) and maize (Vanhees et al., 2020; Vanhees, Schneider, et al., 2021). It is important that the primary root secure adequate water to support the growing seedling, so this root class may benefit from anatomical phenotypes that support penetration of hard soil. Lateral roots emerging from the primary root, as well as seminal root axes and their laterals, may benefit from plastic responses to local soil conditions. However, nodal roots must always pass through the epipedon, which may be dry and hard, on their way to deeper soil domains. Therefore, nodal root axes should be nonplastic; they should ignore local soil conditions including hardness, since they must penetrate the topsoil to benefit the plant. Lateral roots arising from nodal roots could again be plastic in response to local soil impedance to optimally exploit soil resources.
4. *Developmental regulation of root plasticity in dicot crops*: The annual dicotyledonous root system is dominated by one or more orders of lateral roots emerging from a scaffold of axial roots consisting of the primary root in addition to dominant basal roots or early lateral roots in some species. We propose that the elongation of axial root classes should be relatively insensitive to local soil conditions, as they determine the overall architecture and hence soil exploration of the root system, while lateral root emergence and elongation should be plastic to exploit local variation in water availability and soil impedance. Secondary growth of axial roots, or the radial thickening of roots as they age, should ideally display a plastic response to edaphic conditions. In soils with distinct domains of high penetration resistance, the capacity to reallocate resources from root thickening to root elongation would allow for the proliferation of root length in less compacted horizons with greater water and nutrient availability (Strock & Lynch, 2020).

### 5.4 Root ideotypes for carbon sequestration

A significant portion of carbon fixed by plants is deposited into the soil by roots through growth, exudation and associations with soil organisms (Farrar et al., 2003; Jones et al., 2004; Lambers et al., 2002). Root phenotypes that increase the sequestration of atmospheric carbon would not only help to combat climate change but would also serve to alleviate soil degradation by stabilizing aggregates, improving water and nutrient retention, increasing nutrient cycling and sustaining the rhizosphere community. While there is debate over the length of time carbon is stored in the soil currently (Hobley et al., 2014), it is well established that the stabilization of soil organic matter is influenced by both abiotic and biotic factors and that roots contribute a greater fraction to the soil than above‐ground tissues (Ghafoor et al., 2017; Rasse et al., 2005). Ultimately, the persistence of carbon deposited by roots is largely a function of the vertical distribution of the root system (Gill et al., 1999), due to soil conditions that limit the activity of microorganisms in addition to the increased lignin concentrations that are often found in deeper roots (Prieto et al., 2015). Consequently, architectural phenotypes that promote deeper rooting would serve to sequester more enduring carbon in the soil than plants with a shallow distribution of root length (Jobbagy & Jackson, 2000; Kell, 2011, 2012; Lynch & Wojciechowski, 2015; Poirier et al., 2018). Additionally, root phenotypes that improve the metabolic efficiency of soil foraging and improve the growth of above‐ground biomass may, through allometry, produce greater overall root mass and sequestration of carbon in soil.

Compared to carbon derived from above‐ground crop residues, carbon from roots has a longer residence time in the soil (Rasse et al., 2005). Secondary cell wall compounds such as lignin and suberin are abundant in root tissue and have been shown to degrade more slowly than other cell wall compounds like cellulose and hemicellulose (Berg & McClaugherty, 2003; Kogel‐Knabner, 2002; Yue et al., 2016). For example, the suberin content of roots has been identified as one of the most influential components stabilizing soil organic matter (Poirier et al., 2018). Consequently, we propose that dicotyledonous roots that have more secondary growth (Strock & Lynch, 2020) or monocotyledonous roots with thicker cell walls and greater lignin or suberin content (Schneider et al., 2021) could greatly increase the quantity and longevity of carbon deposited into the soil. Finally, root systems with greater exudation or interactions with soil organisms may further contribute to the total quantity of carbon added to the soil. Overall, the ideal root ideotype for sequestering more atmospheric carbon and improving soil C content should have an architecture that promotes deep distribution of root length, anatomical composition with higher concentrations of lignin or suberin per unit mass of tissue and augmented exudation.

## 6 FUTURE DIRECTIONS

### 6.1 The value of considering whole plants in whole soils

We have adopted a ‘whole plant in whole soil’ approach to this problem, which we feel represents a useful complement to much of the existing literature, which primarily focuses on either field‐scale responses of crops to soil management or responses of individual root axes to mechanical impedance. For example, a root axis capable of penetrating a hard soil layer may be seen as advantageous within the context of a single root axis, but may be disadvantageous within the context of an entire plant allocating resources to many root axes exploiting both hard and soft soil domains. Similarly, as we propose here, useful phenotypes may differ among root classes, may vary with time and may vary with soil environments. Additionally, it is important to consider plant responses in natural versus controlled environments. Studies of mechanically impeded soils in controlled growth conditions often involve repacking soil in pots, which changes both the soil bulk density and soil structure including pore size and connectivity. Changes in soil structure, including pore characteristics and air permeability, have significant effects on root growth (Lipiec & Hatano, 2003). Soil structure, including pore size and connectivity, should be considered as an important mechanism for deep rooting in hard soils and considered when interpreting results from controlled experiments. The morphology of whole soils, with horizons having distinct characteristics, is also important to consider, and yet is often lacking from controlled environment studies.

### 6.2 The value of in silico approaches

The vast array of potential root phenotypes interacting with the wide range of potential soil environments results in a high dimensional parameter space that exceeds the capabilities of empirical research. This is especially true given the stochasticity of weather, and given that some root phenotypes of interest, and future climate and soil scenarios, do not yet exist in nature. This calls for robust in silico tools capable of mechanistically linking root phenotypes to plant fitness in a range of soil environments. Specific capabilities of interest in this context include the ability to model the spatiotemporal dynamics of soil hardness and root growth in drying soils with realistic profiles at a relatively fine scale. Such tools exist and are becoming more capable (Dunbabin et al., 2013). Integration of such models with stand‐ and field‐scale models of crop performance in a season and soil quality over multiple seasons will be facilitated by integration tools for multiscale integration (Benes et al., 2020). Linkage of these with landscape models and climate projections will be useful in guiding crop breeding as soils and the climate continue to change.

### 6.3 The value of considering multiple soil taxa

Most research on root responses to impedance has focused on artificial substrates, on growth media derived from a single soil taxon or in field studies, on one or a few related soil taxa. This is clearly related to the requirements for reproducibility in lab studies and on the logistical challenges of working with multiple soil taxa in the field. A related problem is that the preponderance of relevant research has occurred in ecoregions with active research communities. To address global agricultural challenges, we need to understand plant–soil interactions in a wide range of soil taxa. For example, rooting depth in acid tropical soils has very different connotations than rooting depth in younger temperate soils (Lynch & Wojciechowski, 2015). Plant–soil interactions in degraded soils of developing countries represent an important knowledge gap that is critical for global food security. Indigenous research communities in such regions are often poorly resourced. Plant–soil interactions in a range of soil taxa, including those found in developing regions, merit greater attention.

### 6.4 The value of integrating soil and plant expertise

Support for training and research on plant–soil interactions is often highly competitive and is generally focused on specific subdomains that become bandwagons. A current example is the focus on the rhizosphere microbiome. Prioritization of plant molecular biology at the expense of more integrative and holistic approaches has persisted for several decades, and molecular approaches are increasingly important in soil science as well, as interest in the soil microbiome grows. Deep but narrow specialization, within subdomains and often within spatiotemporal scales, is rewarded by most training, funding and publishing regimes. This represents an obstacle in the context of soil/plant relations, which requires expertise in two distinct fields. Cross‐disciplinary expertise in plant biology and soil science is increasingly rare, as is expertise in soil/plant interactions in the crop breeding community. More integrative, transdisciplinary, team‐oriented approaches are needed to develop future crops suited to future soils.

## CONFLICT OF INTERESTS

The authors declare that there are no conflict of interests.
